# One Dose Is Not Enough: The Beneficial Effect of Corrective COVID-19 Information Is Diminished If Followed by Misinformation

**DOI:** 10.1177/20563051231161298

**Published:** 2023-04-17

**Authors:** Michael Craig, Santosh Vijaykumar

**Affiliations:** Northumbria University, UK

**Keywords:** COVID-19, misinformation, corrective information, debunking, experiment

## Abstract

The World Health Organization (WHO) released a series of mythbuster infographics to combat misinformation during the COVID-19 infodemic. While the corrective effects of such debunking interventions have typically been examined in the immediate aftermath of intervention delivery; the durability of these corrective effects and their resilience against subsequent misinformation remains poorly understood. To this end, we asked younger and older adults to rate the truthfulness and credibility of 10 statements containing misinformation about common COVID-19 myths, as well as their willingness to share the statements through social media. They did this three times, before and after experimental interventions within a single study session. In keeping with established findings, exposure to the WHO’s myth-busting infographics—(a) improved participants’ ratings of the misinformation statements as untruthful and uncredible and (b) reduced their reported willingness to share the statements. However, within-subject data revealed these beneficial effects were diminished if corrective information was presented shortly by misinformation, but the effects remained when further corrective information was presented. Throughout the study, younger adults rated the misinformation statements as more truthful and credible and were more willing to share them. Our data reveal that the benefit of COVID-19 debunking interventions may be short-lived if followed shortly by misinformation. Still, the effect can be maintained in the presence of further corrective information. These outcomes provide insights into the effectiveness and durability of corrective information and can influence strategies for tackling health-related misinformation, especially in younger adults.

## Introduction

Public health agencies have launched digital communication interventions to address misperceptions seeded by the online circulation of COVID-19 misinformation. The severity of the COVID-19 misinformation problem is reflected in the World Health Organization (WHO) labeling it an “infodemic” (Calleja et al., 2021). An integral part of combative strategies is the dissemination of “corrective information,” which involves debunking misleading claims circulating on social media (Bavel et al., 2020). A classic example is the “Mythbusters” intervention by the WHO, a digital resource where infographics are used to address public misperceptions related to a range of COVID-19 misinformation (World Health Organization, 2022). Recent work shows that beliefs in COVID-19 misinformation may be reduced through a single exposure to corrective information (Vijaykumar et al., 2021; Vraga & Bode, 2021). Randomized controlled trials of brief 60-s exposure to corrective infographics have yielded minor positive effects supporting arguments about the scalability of such nimble interventions (Agley et al., 2021). However, how long does the protective effect of a single dose last? What happens if people are exposed to misinformation shortly after a dose of corrective information? Misinformation research indicates that light-touch interventions (such as single corrections, infographics, or “accuracy nudges”) dissipate swiftly, even over a duration of seconds in some cases (Roozenbeek et al., 2021). Thus, comprehending the underpinnings of corrective effects and factors that drive their durability has major implications for implementing fact-checks/accuracy nudges and other light-touch interventions in social media environments.

While studies examining the durability of corrective debunking interventions suggest a finite benefit, prebunking interventions that seek to inoculate audiences before misinformation have shown to confer a longer-lasting effect (2–6 weeks) (van der Linden et al., 2021). Prebunking might be ideal for inoculating the public against misinformation in a general sense, but black swan events like the COVID-19 or even other infectious disease outbreaks like Ebola and Zika arrive under atypical conditions. Specifically, these pertain to unique disease characteristics, minimal understanding of the nature of their impact on human health, and mystery surrounding modes of transmission, all of which create a fertile breeding ground for misinformation to emerge and proliferate. New misinformation content specific to these conditions then emerge and spread, commanding public health agencies to respond swiftly using debunking strategies. Research on debunking political misinformation has demonstrated that the effects of reaffirming truths and retracting falsehoods resulted in participants re-believing the misinformation after a week, suggesting a “continued influence” of misinformation (Swire et al., 2017).

Moreover, the endurance of post-information corrective effects may be strengthened by repeated exposure to corrective information through strategies like booster sessions and weakened by decaying factors like political predispositions and pre-existing attitudes (Carnahan et al., 2021). Understanding the specific mechanisms underpinning these findings allows the development of targeted interventions to reduce misinformation effects. These problems have been investigated less in the public health context, with the COVID-19 pandemic amplifying the need for more research to understand effective debunking strategies.

To achieve this, three primary gaps in our understanding of the durability of corrective information must be addressed. The first involves assessing the durability of the impact of real-world public health communication interventions like the WHO’s infographics. Second, durability assessments need to incorporate the ephemeral and transient nature of the flow of information on social media where users could be exposed to a trove of information, often with competing narratives within minutes. Third, the seemingly changeable impact of age on the durability of corrective effects must be understood. We first discuss the cognitive and behavioral outcomes that corrective information interventions seek to influence and then provide a rationale for focusing on age as a critical individual factor in this process.

## Cognitive and Behavioral Impacts of Corrective Information

Our evaluation of the durability of corrective information interventions like the mythbusters is premised on its ability to steer and sustain three cognitive and behavioral responses in the desired direction: perceived truthfulness, perceived credibility, and intention-to-share the information.

### Perceived Truthfulness

Debunking interventions using corrective information are commonly evaluated based on their ability to shift audience’s beliefs away from misinformation and strengthen their ability to correctly identify the accuracy of these messages. Evaluating the accuracy of the content becomes especially important while engaging with the social media ecosystem where audiences could be exposed to information of various levels or provenance, or “shades of truth” from fully false to partly false and fully true (Lockyer et al., 2021; Wang, 2017). Partly, false content can be especially problematic given that it can entrench beliefs in misinformation and undermine the effectiveness of corrective information (Freelon & Wells, 2020; Southwell et al., 2018). Low levels of knowledge, dependence on heuristic cues like fluency, and reasoning ability can affect the ability to discern between accurate and inaccurate information (Pennycook & Rand, 2021), but the role of repeated exposure to messages is especially important. The illusory truth effect says that people tend to perceive information as truer if they have been exposed to it before (Hassan & Barber, 2021). This means, for instance, that being exposed to the same COVID-19 falsehood arriving via different WhatsApp groups or connections can enhance the truthfulness of misinformation. The criticality of timely dissemination of corrective information is amplified even further in such situations. While some uncertainty remained over relevance of the illusory truth effect in claims that are obviously true or false, recent evidence from a simulated experiment shows its influence persisted across ambiguous and unambiguous statements (Fazio et al., 2019). The magnitude of the effect of repetition in the context of a real-world public health intervention, such as the WHO’s mythbusters is less understood and will be investigated in this study.

### Message Credibility

Assessments about the accuracy of messages (perceived truthfulness), in turn are shown to affect perceptions about its’ credibility (Jung et al., 2016). The perceived credibility of the message is defined as “an individual’s judgment about the veracity of the content of the communication” (Appelman & Sundar, 2016). Four broad categories of factors that can influence the perceived credibility of corrective information, and its potential to persuade audiences away from believing misinformation (E.-J. Lee & Shin, 2021). (1) Message characteristics: Messages that are consistent, as opposed to discordant, with one’s beliefs systems might seem more credible because these are easier to recall and can be used to arrive at a conclusion (Nickerson, 1998; Zhou & Shen, 2022). While evidentiary devices like statistics, graphs and quotes are often included to strengthen the credibility of corrective information, the “truth bias” imposed by these strategies can also be leveraged to spread misinformation (Newman et al., 2012). The frequency with which messages are disseminated could play a critical role in enhancing their perceived credibility, as suggested earlier by the “illusory truth” bias. In other words, if repeated exposure to misinformation can enhance the believability of false claims, it is plausible that a similar strategy could be used with corrective information for beneficial effects. However, corrective information by public health agencies like the WHO’s mythbusters are often online resources in stasis on their website with no possible determination about how frequently audiences are exposed to them. One of the focal points of this study is to determine if a single exposure can bear lasting effects. (2) Source characteristics: Specific attributes of information sources have proved useful in strengthening to benefits of corrective interventions as they provide important social cues (Ecker et al., 2022) . For instance, corrective interventions delivered by government authorities and health experts minimize misinformation belief to a greater extent than social peers (van der Meer & Jin, 2020). Messages seem truer when delivered by credible, as opposed to non-credible sources, or sources who seem familiar, attractive, and powerful (Briñol & Petty, 2009; Nadarevic et al., 2020). However, people’s inattentiveness and forgetfulness could undermine source effects on credibility judgments with some studies showing that people can discern the veracity of (mis)information irrespective of the source (Vijaykumar et al., 2021). Based on this evidence, our experimental stimuli mention the source of the mythbusters (WHO) but measures the perceived credibility of the message as opposed to the institution. (3) Channel: Channel considerations pertain to the modality (images vs text), synchronicity (delivered in real time vs delivered with a delay), and medium (traditional media vs social media) (Lee & Shin, 2021). Of most relevance to this study is consistent evidence that images possess greater persuasive power than simply text and are perceived to be more informative and useful (Lee & Shin, 2021; Lee et al., 2022). Building on this strand, mythbuster infographics disseminated by the WHO consistently minimized COVID-19 misperceptions (Vijaykumar et al., 2021; Vraga & Bode, 2021). (4) Individual factors: While several individual characteristics, such as knowledge and numerical literacy render individuals vulnerable to misbelieving misinformation to be credible (Roozenbeek et al., 2020), our study seeks to shed further clarity on the inconclusive debates around the role of age. Our arguments are presented at the end of this sub-section.

### Intention-to-Share

In COVID-19 and non-COVID-19 contexts, evidence shows that messages that are perceived to be credible are also more likely to be shared (Song et al., 2023; Stefanone et al., 2019). Sharing behavior underpins the extent to which extent to which information spreads or goes “viral” on social media, potentially influencing behavioral intentions (Alhabash & McAlister, 2015). Viral content can quickly reach and influence greater numbers of audiences, with dangerous or beneficial effects shared more widely and quickly depending on the nature of the content. For instance, health misinformation about COVID-19 vaccines that goes viral on social media can infuse doubts about the side effects of the vaccine leading to vaccine hesitancy and potentially vaccine refusal (Dror et al., 2020). While sharing accurate information potentially confers greater societal benefits, research has shown that misinformation is shared more widely and quickly possibly because of its novelty and ability to elicit emotional reactions (Vosoughi et al., 2018). Among health communication strategies that can trigger further dissemination by audiences, recent research shows that infographics trigger greater sharing intentions especially while messaging about health issues related to proximal health behaviors or outcomes (e.g., a flu shot) (incomplete) and can thus be especially relevant during infectious disease outbreaks (Lee et al., 2022). Previous work has also demonstrated that that the WHO’s mythbusters infographics can positively affect sharing intentions related to accurate misinformation (Vijaykumar et al., 2021). We build on this investigate how sharing intentions fluctuate in the face of repeated exposure to misinformation or corrective information.

## Age and Misinformation

Of the various individual level factors that drive vulnerability to misinformation, the evidence surrounding the relationship between age and misinformation commands is particularly conflicting. For instance, older adults (over 65 years of age) were seven times more likely to share political fake news as opposed to younger adults aged 18–29 (Guess et al., 2019). These findings are explained by lower levels of digital media literacy among older adults and the detrimental effect of age-related memory decline on increased susceptibility to the “illusion of truth” effect (where repeated exposure to a false claim can make it seem like the truth). Similar explanations have been provided for findings which suggest that older white men are more likely to be engaged with fake news sources (Grinberg et al., 2019). Analyses of media consumption patterns show that greater television consumption by older adults (55+) might expose them to ordinary bias and agenda setting by the mainstream media (Allen et al., 2020). The dependence on information they are familiar with (fluency), challenges with source recall and difficulties with detecting deception are other reasons why older people may be vulnerable to misinformation (Brashier & Schacter, 2020).

However, other studies have found weak associations between older age and susceptibility to COVID-19 misinformation in four of five countries (the only exception being Mexico) (Roozenbeek et al., 2020). A randomized online survey experiment of the effectiveness of the WHO’s mythbuster infographics found that younger adults (18–35) demonstrated stronger beliefs in misinformation than participants 55 years or older (Vijaykumar et al., 2021). These findings are partly explained by the ability of older adults to accumulate facts over time and evaluate the veracity of new information based on how it aligns with their general knowledge (Brashier & Schacter, 2020). An experiment testing the illusory truth effect between younger and older adults finding minimal differences between the two groups (Mutter et al., 1995; Parks & Toth, 2006). In sum, the evidence around the effect of age on vulnerability to misinformation is mixed with divergent findings across political misinformation, health misinformation, and more generic misinformation like trivia.

### Study Aims and Hypotheses

To this end, we asked younger and older adults to rate the truthfulness and credibility of ten statements containing misinformation about different COVID-19 myths, as well as their willingness to share the statements through social media. They did this on three occasions within a single session: (a) on entering the study (Baseline), (b) following exposure to ten corrective infographics developed by the WHO, one per misinformation statement (Intervention 1), and then (c) after exposure to 10 WhatsApp messages (Intervention 2). Five of the WhatsApp messages contained *misinformation* relating to five of the statements, and the remaining five contained *corrective information* relating to the other five statements.

In keeping with existing literature, we predicted that exposure to the debunking infographics (Intervention 1) would improve participants’ ratings of the misinformation statements as untruthful and uncredible and reduce their willingness to share the statements through social media. Critical to the current study, should the benefit of corrective information be abated by subsequent misinformation, we hypothesized the effect of Intervention 1 should be reduced, at least somewhat, in response to Intervention 2, but *only* for the five statements that receive WhatsApp messages containing misinformation. For the five statements that revived a second “dose” of corrective information in Intervention 2, we predicted that the benefit of Intervention 1 should be maintained, and possibly improved, should two “doses”—in proximity—be better than one. Finally, if older adults are less susceptible to COVID-19 misinformation, they should correctly rate the misinformation statements as less truthful and credible and be less willing to share them. Because of this, intervention effects may be less pronounced in this population.

## Methods

### Participants

**Figure 1.**
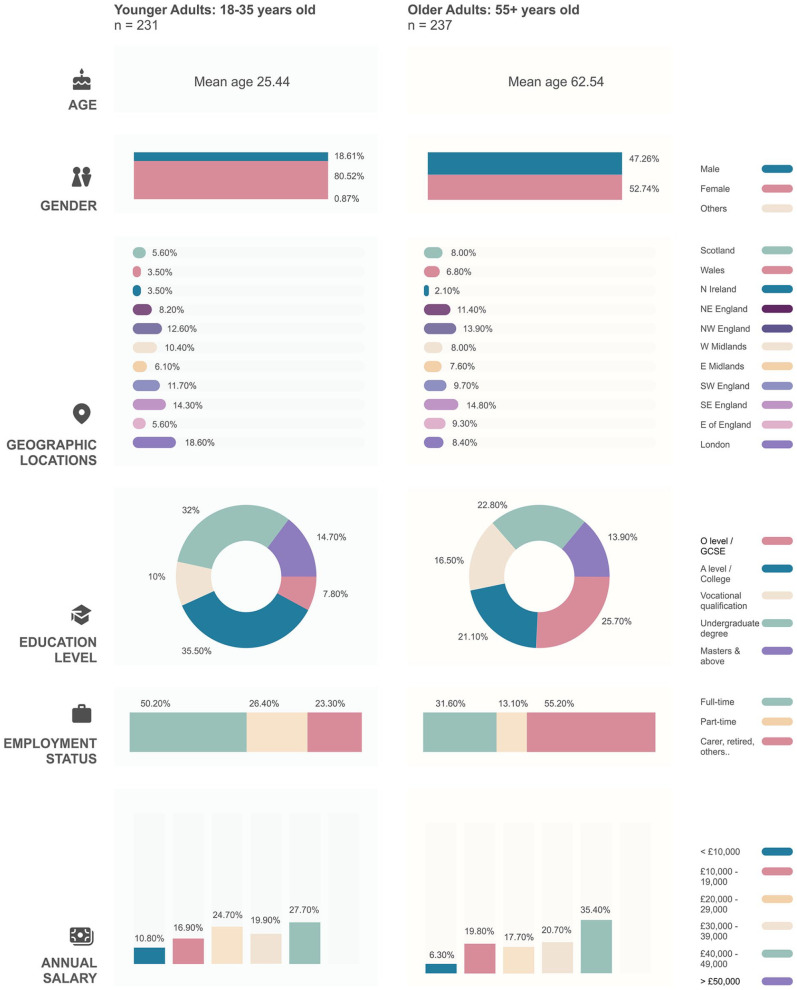
Participant demographics. The figure summarizes participant demographic information for our younger (*n* = 231) and older (*n* = 237) adult groups. Details are shown regarding participants’ ages, gender, geographic location in the United Kingdom, highest education level, current employment status, and annual salary.

An a priori analysis of the sample required was conducted using G*Power (Version 3.1.9.7). To detect a difference between age groups with a medium effect size (*d* = 0.50), 0.05 probability of error, and 0.90 power, a total sample of 172 participants were required (*n* = 86 per age group). We exceeded this target through the recruitment of 231 younger adults aged 18–35 years old (43 males, 186 females, 2 other; *M*_age_ = 25.44 years, *SD* = 5.13 years; age range = 18–35 years) and 237 older adults aged 55 years old and above (112 males, 125 females; *M*_age_ = 62.54 years, *SD* = 6.12 years; age range = 55–81 years). Categorization of younger and older adults as those aged 18–35 years and 55+ years old, respectively, was based on commonly used age ranges in psychological and biomedical literature (cite). These individuals were recruited through Qualtrics’ panel of survey respondents. In addition, participants were required to fit our criteria for younger and older adults (see above), live in the United Kingdom, and be WhatsApp users aware of COVID-19. Aside from age (younger vs older), we had no a priori predictions surrounding the contribution of other demographic factors, for example, gender and employment status, and thus did not control for these factors in our recruited sample. Figure 1 provides a visual overview of participant demographics, which were broadly representative of the general population. Data collection commenced on 15 December 2020 and culminated on 10 March 2021. Throughout this time, the United Kingdom remained under relatively severe “lockdown” restrictions, including mask-wearing, social distancing, and restricted mixing of households. All participants provided written informed consent to participating in the study before responding to the survey questions. Given the nature of the study, when being debriefed, participants were directed toward truthful COVID-19 information about the topics covered in the study. The study was approved by the Faculty Ethics Committee at a large university in England (Ref: 120.1520).

### Design

To examine whether the beneficial effect of corrective COVID-19 interventions can withstand subsequent misinformation in younger and older adults, we employed a repeated measures (RMs) design with between-subject factor *age group* (younger adults vs older adults) and within-subject factors *time of test* (Baseline vs Intervention 1 vs Intervention 2) and *truthfulness of information presented in Intervention 2* (corrective information vs misinformation). The study took place in a single session and was delivered online through the research platform Qualtrics.

### Materials

From the WHO’s COVID-19 mythbuster webpage, which offers corrective infographics to debunk prevalent COVID-19 misinformation online, we selected five themes: *therapeutics*, *environment*, *behavior*, *foodstuffs*, and *transmission*. **Ten infographics** (two per theme) were selected from the WHO’s website. Within the remit of the limited number of infographics available, the two infographics selected for each theme were matched as closely as possible on their topic and content, for example, that experiencing *cold temperatures* and *hot temperatures* can cure COVID-19 (*environment* theme). These infographics were presented in the Intervention 1 phase—see the “Procedure” section.

Based on these ten infographics, we developed corresponding **misinformation statements**. For example, for an infographic tackling the myth that garlic can cure COVID-19 (*foodstuffs* theme), the following statement was prepared: “Garlic can cure me of the Coronavirus (COVID-19).” Similarly, for an infographic tackling the myth that COVID-19 can be transmitted through 5G networks (*transmission* theme), the following statement was developed: “Viruses like Coronavirus (COVID-19) can be spread through mobile networks like 5G.” These ten misinformation messages were presented to participants in each phase of our study. They were asked to rate the truthfulness and credibility of the statements and their willingness to share them through social media—see the “Procedure” section.

Further to the above, based on the ten misinformation statements and linked corrective infographics, we developed ten graphics designed in the form of **forwarded WhatsApp messages**. Each WhatsApp message related to one of the ten misinformation statements. Critical to the purpose of the current study, these messages contained either (a) corrective information (total = 5) or (b) misinformation (total = 5). For each of the five themes of misinformation, one WhatsApp message (e.g., *hot temperatures cure COVID-19*) contained correct information, for example, “research shows that hot temperatures do not cure COVID-19.” The other WhatsApp message (e.g., *cold temperatures cure COVID-19*) contained misinformation, for example, “research shows that hot temperatures can cure COVID-19.” These graphics were presented to participants in the Intervention 2 phase—see the “Procedure” section. All materials are available through the project’s OSF site: https://osf.io/4qm7y/.

The choice of WhatsApp-based stimuli for this study was based on several reasons. WhatsApp is the most used messaging service in the United Kingdom with more than 40 million users and was one of the global vectors of misinformation during the COVID-19 pandemic (cite). Resultantly, several organizations, including the WHO and the International Fact Checking Network, launched WhatsApp-based interventions like tiplines to combat the spread and impact of misinformation.

### Measures

To establish whether the beneficial effect of corrective COVID-19 information is resilient against exposure to subsequent misinformation, we employed three dependent variable measures concerning misinformation belief. These three measures were applied in each phase of our study: Baseline, Intervention 1 (corrective information), and Intervention 2 (correct information vs misinformation).

First, we applied a measure of perceived **truthfulness**, where participants are required to “rate the truthfulness” of information on a scale from 1 = *not at all* to 9 = *very*. This measure was based on methods investigating the perceived accuracy of health-related messages (Carey et al., 2020), and which was updated recently for the context of COVID-19 misinformation (Vijaykumar et al., 2021).

Second, a measure of message **credibility** was employed (Appelman & Sundar, 2016). This scale-based measure asks participants to rate how well (from 1 = *very poorly* to 9 = *very* well) three adjectives describe communication content: *accurate*, *authentic*, and *believable*. We amended the scale from a 7- to 9-point scale for the current study. Given that scale, reliability analyses suggest this three-item measure has high internal reliability (α = 0.87) (Appelman & Sundar, 2016), we averaged responses from the three sub-scores into a single score (min. score = 1, max. score = 9) for analyses. Cronbach’s analyses confirmed high internal reliability across the three scale items in the current study (α > 0.9 in all instances).

Third, given the importance of misinformation dissemination, a **sharing** measure was used to explore participants’ willingness to share messages containing misinformation through social media. Specifically, based on existing methods (Lee & Ma, 2012), participants are asked how likely they would *intend, expect*, and *plan* to share content through social media. A rating on a 5-point scale from 1 = *highly unlikely* to 5 = *highly likely* was collected for each verb. Cronbach’s analyses confirmed high internal reliability across the three scale items in the current study (α > 0.9 in all instances). Because of this, we averaged responses from the three sub-scores into a single score (min. score = 1, max. score = 5) for analyses.

### Procedure

Our experimental procedure was inspired by research investigating the correction of misinformation (Lewandowsky & van der Linden, 2021; Vijaykumar et al., 2021; Vraga & Bode, 2021) and memory paradigms used to examine the effect of within-subject manipulations on memory accuracy during reconsolidation (Hupbach et al., 2007; Przybyslawski & Sara, 1997). Participants were informed that they were participating in a study investigating how we make judgments about COVID-19 information found online. The procedure comprised three phases and took place in a single session: Baseline, Intervention 1, and Intervention 2. During the **Baseline** phase, participants were presented sequentially ten misinformation messages relating to prevalent COVID-19 myths identified by the WHO (see the “Materials” section). For example, “Garlic can cure me of the Coronavirus (COVID-19).” For each statement, participants were asked to rate the truthfulness and credibility of the messages. Their willingness to share the messages through social media was also probed. There was no time limit to respond. These measurements provided a pre-intervention baseline for relative comparison to establish post-intervention effects.

In the subsequent **Intervention 1** phase, participants were presented corrective COVID-19 information in the form of the WHO’s COVID-19 mythbuster infographics (see the “Materials” section). Ten infographics were presented, one concerning each topic covered in the ten misinformation statements (e.g., garlic cures COVID-19). The infographics were presented sequentially and in a random order, each for 30 seconds (total duration = 5 min). This fixed duration ensured all participants received identical treatment and exposure to corrective stimuli, opposed to self-paced exposure as used in related work (Basol et al., 2021). After exposure to the corrective information, participants rated the truthfulness and credibility of the same ten randomly ordered misinformation statements for a second time as presented in the Baseline phase. They were also again asked to rate their willingness to share the statements. We did this to establish whether, as in previous work, exposure to corrective information positively affects participants’ treatment of misinformation.

Following this, in the **Intervention 2** phase, participants were presented ten WhatsApp messages, each concerning one of the topics covered in the ten misinformation statements (see the “Materials” section). Critical to our hypotheses, five of the messages contained *misinformation* and five contained *corrective information*. This within-subject manipulation enabled us to examine whether the possible benefit of corrective information in the Intervention 1 phase is abated by subsequent misinformation. If so, a corrective effect from Intervention 1 should be reduced, at least somewhat, in response to Intervention 2, but only for the five statements that receive misinformation in the WhatsApp messages. For the reasons explained above, WhatsApp messages were ordered randomly and presented sequentially for 30 s (total duration = 5 min). After exposure to the WhatsApp messages, participants rated the truthfulness and credibility of the same ten randomly ordered misinformation statements presented in the Baseline and Intervention 1 phases for a third and final time. They were also again asked to rate their willingness to share the statements.

### Statistical Analyses

For the Baseline, Intervention 1, and Intervention 2 phases, mean truthfulness, credibility, and sharing scores were computed for (a) the five COVID-19 topics that received corrective information in Intervention 2 and (b) the five COVID-19 topics that received misinformation in Intervention 2. Data were analyzed using IBM SPSS Statistics (Version 26). Truthfulness, credibility, and sharing measures were investigated using individual RM analyses of variance (ANOVAs) with between-subject factor age group (younger adults vs older adults) and within-subject factors time of test (Baseline vs Intervention 1 vs Intervention 2) and Intervention 2 manipulation (corrective information vs misinformation). Pairwise comparisons were used to examine within-subject changes in responses from one time point to another (effect of Intervention 1: Baseline vs Intervention 1). They were also used to compare—within each age group—mean scores for each study phase, for example, comparison of mean truthfulness scores recorded at the Intervention 2 phase for items that received corrective information in Intervention 2 versus items that received misinformation in Intervention 2. Bonferroni corrections were applied to correct for multiple comparisons.

## Results

### Perceived Truthfulness

**Figure 2.**
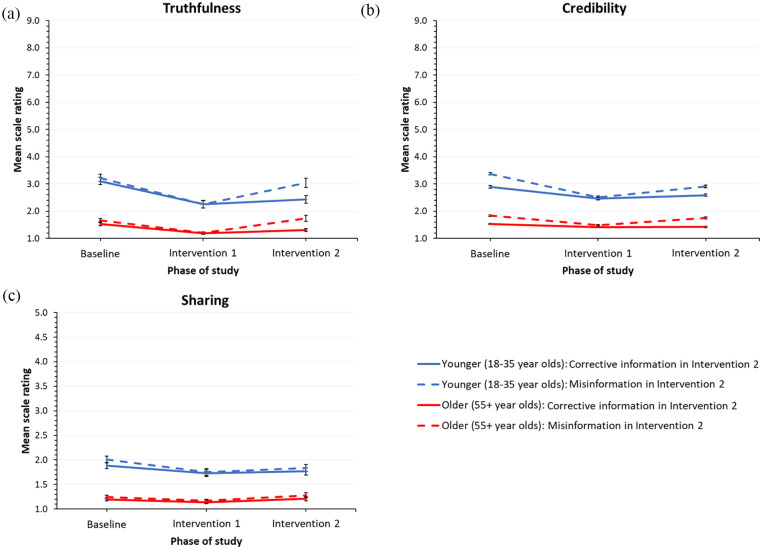
Performances in perceived truthfulness, message credibility, and sharing intention measures. The line graphs show mean scores for the truthfulness, credibility, and sharing measures from each study phase broken down by between-subject factor age (younger vs older) and within-subject factor Intervention 2 manipulation (corrective information vs misinformation). Blue lines show data from younger adults, and red lines show data from older adults. Solid lines refer to data for statements presenting truthful information in Intervention 2 (total = 5), and dashed lines refer to data for statements presenting novel misinformation in Intervention 2 (total = 5). In all cases, a lower score reflects superior performance. Error bars show the standard error of the mean. Post hoc pairwise comparisons conducted individually for younger and older adults revealed significant declines in scores between Interventions 1 and 2 testing times for items that received corrective information in Intervention 1 and misinformation in intervention 2 (all *p* < .005).

Figure 2a shows mean truthfulness scores for each study phase broken down by age group (younger vs older) and our Intervention 2 manipulation (corrective information vs misinformation). We observed a significant main effect of time of test (*F*(2,932) = 82.305, *p* < .001, ηρ² = .150) because there was an improvement in ratings following the presentation of corrective information in Intervention 1 and worsening in response to Intervention 2, predominantly for items that received misinformation in this study phase. This was reinforced through a significant effect of our Intervention 2 manipulation (*F*(1,466) = 33.347, *p* < .001, ηρ² = .109), where those who received misinformation in Intervention 2 generally performed poorer than those who received corrective information in the same study phase. A significant interaction between time of test and our Intervention 2 manipulation was observed (*F*(2,932) = 38.358, *p* < .001, ηρ² = .076) because the effect of this intervention (corrective information vs misinformation) was largely restricted to the final phase of our study (see Figure 2a). Pairwise comparisons revealed that item subset scores did differ significantly during the Baseline phase (younger: *t*(230) = –2.046, *p* = .042; older: *t*(236) = –3.374, *p* = .001), but were matched following presentation of corrective infographics in Intervention 1 (younger: *t*(230) = .159, *p* = .874; older: *t*(236) = –.299, *p* = .765). A negative change in scores for items that received misinformation in Intervention 2 resulted in a significant difference between item subset scores in this phase (younger: *t*(230) = –6.173, *p* < .001; older: *t*(236) = –4.810, *p* < .001).

Throughout the study, older adults outperformed younger adults in the truthfulness measure (*F*(1,466) = 87.732, *p* < .001, ηρ² = .158), where the former performed near ceiling. A significant interaction between time of test and age was observed (*F*(2,932) = 16.347, *p* < .001, ηρ² = .027) because the effect of our Intervention 1 manipulation was more pronounced in younger adults. However, this was somewhat driven by near ceiling effects in older participants, that is, there was little room for them to improve. There was no significant interaction between age and our Intervention 2 manipulation (*F*(1,466) = 0.687, *p* = .408, ηρ² = 001), indicating that the effect of corrective information vs. misinformation was comparable in younger and older adults. Furthermore, we found no three-way interaction between age, time of test, and Intervention 2 manipulation (*F*(2,932) = 1.793, *p* = .167, ηρ² = .004). All significant effects from the RM ANOVA remained after controlling for multiple comparisons (Bonferroni-corrected *p*-value = .007).

When gender was included as a covariate in the RM ANOVA, no significant findings changed, and overall trends remained. We did, however, observe a significant main effect of gender (*F*(1,465) = 16.073, *p* < .001, ηρ² = .033) because males performed poorer in this measure. There were no two- or three-way interactions between gender and our other factors (all *p*s > .112), indicating that the effect of age, time, and Intervention 2 manipulation were comparable across genders.

### Message Credibility

Figure 2b shows mean credibility scores for each study phase broken down by age group (younger vs older) and our Intervention 2 manipulation (corrective information vs misinformation). We observed a significant main effect of time of test (*F*(2,932) = 31.912, *p* < .001, ηρ² = .064) because there was an improvement in ratings following the presentation of corrective information in Intervention 1 and worsening in response to Intervention 2, predominantly for items that received misinformation in this study phase. This was reinforced through a significant effect of our Intervention 2 manipulation (*F*(1,466) = 119.552, *p* < .001, ηρ² = .204), where those who received misinformation in Intervention 2 generally performed poorer than those who received corrective information in the same study phase. A significant interaction between time of test and our Intervention 2 manipulation was observed (*F*(2,932) = 26.744, *p* < .001, ηρ² = .054) because the effect of this intervention (corrective information vs misinformation) was largely restricted to the final phase of our study (see Figure 2b). Pairwise comparisons revealed that item subset scores did differ significantly during the Baseline phase (younger: *t*(230) = –8.376, *p* < .001; older: *t*(236) = –8.053, *p* < .001), but were matched following presentation of corrective infographics in Intervention 1 (younger: *t*(230) = –1.412, *p* = .159; older: *t*(236) = –1.873, *p* = .062). A negative change in scores for items that received misinformation in Intervention 2 resulted in a significant difference between item subset scores in this phase (younger: *t*(230) = –5.180, *p* < .001; older: *t*(236) = –4.541, *p* < .001).

Throughout the study, older adults outperformed younger adults in the credibility measure (*F*(1,466) = 66.128, *p* < .001, ηρ² = .124), where the former performed near ceiling. A significant interaction between time of test and age was observed (*F*(2,932) = 7.544, *p* < .001, ηρ² = .016) because the effect of our Intervention 1 manipulation was more pronounced in younger adults. There was no significant interaction between age and our Intervention 2 manipulation (*F*(1,466) = 0.860, *p* = .835, ηρ² = .002), indicating that the effect of corrective information versus misinformation was comparable in younger and older adults. Furthermore, we found no three-way interaction between age, time of test, and Intervention 2 manipulation (*F*(2,932) = 2.404, *p* = .091, ηρ² = .005). All significant effects from the RM ANOVA remained after controlling for multiple comparisons (Bonferroni-corrected *p*-value = .007).

When gender was included as a covariate in the RM ANOVA, no significant findings changed, and overall trends remained. We did, however, observe a significant main effect of gender (*F*(1,465) = 9.911, *p* = .002, ηρ² = .021) because males performed poorer in this measure. There was also a significant interaction between gender and our Intervention 2 manipulation (*F*(1,465) = 8.597, *p* = .004, ηρ² = .018) because the effect of our manipulation was more pronounced in males though, like the interaction between age and our Intervention 2 manipulation, this was at least partially driven by females performing closer to ceiling and thus having less room for improvement. No other interactions were significant (all *p* > 300).

### Sharing Intention

Figure 2c shows mean sharing scores for each study phase broken down by age group (younger vs older) and our Intervention 2 manipulation (corrective information vs misinformation). In keeping with our other measures, we observed a significant main effect of time of test (*F*(2,932) = 16.330, *p* < .001, ηρ² = .034) because there was an improvement in ratings following the presentation of corrective information in Intervention 1 and worsening in response to Intervention 2, predominantly for items that received misinformation in this study phase. This was reinforced through a significant effect of our Intervention 2 manipulation (*F*(1,466) = 47.706, *p* < .001, ηρ² = .093), where those who received misinformation in Intervention 2 generally performed poorer than those who received corrective information in the same phase. A significant interaction between time of test and our Intervention 2 manipulation was observed (*F*(2,932) = 6.752, *p* = .001, ηρ² = .014) because the effect of this intervention (corrective information vs misinformation) was largely restricted to the final phase of our study (see Figure 2c). Pairwise comparisons revealed that item subset scores differed significantly during the Baseline phase (younger: *t*(230) = –5.241, *p* < .001; older: *t*(236) = –3.376, *p* = .001), Intervention 1 phase (younger: *t*(230) = –1.300, *p* = .195; older: *t*(236) = –2.625, *p* = .009), and Intervention 2 phase (younger: *t*(230) = –2.768, *p* = .006; older: *t*(236) = –3.389, *p* < .001), though the magnitude of the difference was more pronounced following our Intervention 2 manipulation (see Figure 2c).

Throughout the study, older adults outperformed younger adults in the sharing intention measure (*F*(1,466) = 72.654, *p* < .001, ηρ² = .135), where the former performed near ceiling. A significant interaction between time of test and age was observed (*F*(2,932) = 7.745, *p* < .001, ηρ² = .016) because the effect of our Intervention 1 manipulation was more pronounced in younger adults. There was no significant interaction between age and our Intervention 2 manipulation (*F*(1,466) = 1.202, *p* = .273, ηρ² = .003), indicating that the effect of corrective information versus misinformation was comparable in younger and older adults. We did find a three-way interaction between age, time of test, and Intervention 2 manipulation (*F*(2,932) = 3.889, *p* = .049, ηρ² = .008), but this effect did not survive correction for multiple comparisons (Bonferroni-corrected *p*-value = .007). All other effects remained significant.

When gender was included as a covariate in the RM ANOVA, no significant findings changed, and overall trends remained. We did, however, observe a significant main effect of gender (*F*(1,465) = 7.551, *p* = .006, ηρ² = .016) because males performed poorer in this measure. There were no two- or three-way interactions between gender and our other factors (all *p* > .661), indicating that the effect of age, time, and Intervention 2 manipulation were comparable across genders.

## Discussion

The durability of corrective information by public health agencies on misinformation beliefs among social media users has seldom been investigated. For example, Vraga and Bode (2021) investigated the efficacy of WHO’s infographics similar to the stimuli used in our study but focused on placement and source and not on durability. Meanwhile, Basol et al. (2021) found that COVID-19 infographics were less effective than prebunking inoculation strategies to improve people’s confidence in spotting misinformation and reduce their willingness to share it. However, they used UNESCO infographics which contained more generic educational content than specific, topic-specific debunking content in our stimuli.

In keeping with existing literature, we found that exposure to corrective information—the WHO’s “Mythbuster” infographics—improved participants rating of misinformation statements as untruthful and uncredible. It also reduced their willingness to share the statement through social media. However, our data suggest that this beneficial effect of a “single dose” of corrective information is short-lived *if* it is followed shortly by exposure to misinformation (Intervention 2). Critically, this effect was observed only for items where misinformation was presented in Intervention 2: exposure to further corrective information (i.e., a “double dose”) did not result in *further* improvements. Still, it did maintain the benefit of a single dose of corrective information. These findings reveal that the lifespan of a single dose of corrective information may not be sufficient to deliver long-lasting protection against COVID-19 misinformation. Furthermore, outcomes may be of particular importance for younger adults, who demonstrated higher misinformation belief and willingness to share throughout our study. We discuss these findings and possible explanations in turn.

The benefit of corrective information in the Intervention 1 phase resonates with established effects following the debunking of misinformation, including about COVID-19 (Kreps & Kriner, 2020; van der Linden et al., 2021; Vijaykumar et al., 2021). In addition, this work has included observance of corrective effects following the WHO’s infographics application (Basol et al., 2021). Pinpointing the drivers of this positive change is difficult to establish in our design but might be explained straightforwardly through the influence of the information presented on attitudes toward misinformation. This explanation may also account for the diminished benefit seen in Intervention 2 for the subset of statements for which misinformation was presented. Inherent differences between item subsets are unlikely to explain the within-subject effect of our Intervention 2 manipulation. Despite some initial differences between item subsets in the Baseline phase, the corrective effect of infographics in the Intervention 1 phase acted as a “leveller”: truthfulness, credibility, and sharing scores were well-matched when probed in the Intervention 1 phase, which immediately preceded our within-subject Intervention 2 manipulation. Nevertheless, to rule out the contribution of item-by-item effects, we acknowledge that it would be advantageous to replicate our findings using a set of statements that were closely matched in the Baseline phase. Still, it is important that irrespective of any differences between items, other than the effect of age group, all effects reported reflected within-subject changes that were in response to our experimental manipulations.

An alternative explanation for the observed effects is that our experimental design affected the content of retained memories pertaining to the common COVID-19 myths. This possibility is in keeping with evidence demonstrating that memories are not fixed and can be altered/updated (for better or worse) through exposure to subsequent information shortly following their initial acquisition and subsequent recall, which influence consolidation and reconsolidation processes, respectively (Dudai & Eisenberg, 2004; Loftus, 2005; Spiers & Bendor, 2014). Even subtle cues, less prominent than used in the current study, are found to re-enter memories into a labile state (Hupbach et al., 2007). Such memory studies inspired our experimental design. Therefore, it is possible that the (mis)information presented in the current study updated existing traces, which was detected in subsequent questioning. Indeed, given that questioning often occurred several minutes post-intervention exposure, this suggests that the effects reported in the current study did not dissipate rapidly but remain *at least* over the time course of minutes. This duration may be further indicative of a contribution of memory to our findings. Our design does not allow us to confirm this but may offer inspiration for future work. Indeed, the contribution of memory mechanisms to misinformation is noted as a promising area of investigation (van der Linden et al., 2021).

Further to these possibilities, other factors may have contributed to our findings, and we cannot rule out the contribution of demand characteristics. But the likelihood of extensive influence of experimenter influence is low given that participants were (a) unaware of the exact purpose of the study, (b) not informed whether presented information was truthful or not, and (c) provided ratings of truthfulness, credibility, and sharing (in most cases) several minutes after exposure to (mis)information in Interventions 1 and 2. Had stimuli exposure and ratings been collected simultaneously, this may be more likely. Therefore, we propose influence of presented information on attitudes, and possible contributions in memory, are more likely explanations.

It is of interest that there was no *extra* benefit in the second intervention phase for misinformation statements that received further corrective information. This might suggest that two “doses” of corrective information within minutes of one another have no added benefit over a single dose. While this is possible, our data cannot account for differences in the strength of the effect that may influence its durability. Thus, while our study offers new insights into the limited and temporary effectiveness of a single dose of corrective information, we cannot make inferences about the durability of two doses, other than demonstrating no negative effect of a second dose, even when presented in a different medium to the first.

How can the striking effect of age in misinformation belief and willingness to share misinformation be explained? Heightened misinformation belief and willingness to share misinformation in younger adults is in keeping with recent findings, but data are mixed, and other misinformation research suggests an effect of age in the opposing direction (Allen et al., 2020; Grinberg et al., 2019; Guess et al., 2019; Roozenbeek et al., 2020; Vijaykumar et al., 2021). These findings may be influenced by a broad range of factors, including political ideology, religiosity, and social ideology, which we did not measure here but are known to contribute to misinformation belief (Grinberg et al., 2019; Swire et al., 2017). In addition, behaviors surrounding social media use may also have contributed. Specifically, greater use of social media platforms, particularly news-seeking behaviors (Edgerly, 2017), may have resulted in our younger adults being exposed to more corrective information, and also misinformation. Indeed, this population are reported to be more likely to see and share COVID-19 disinformation (Crime and Security Research Institute, 2020; Herrero-Diz et al., 2020; Ofcom, 2019).

The effects of our interventions were less pronounced in older individuals partially because they performed near ceiling and demonstrated very little belief in the misinformation statements. In addition, older adults’ may rely on their more extensive knowledge and critically evaluate new information (Umanath & Marsh, 2014). The same may hold in the current study. A further consideration is that sampling only WhatsApp users may have resulted in the recruitment of digital and media literate older adults who are experienced in fact-checking online. If so, our sample may not be truly representative of the older adult population. Ceiling effects meant a reduced capacity to observe a benefit of corrective information in our older sample. Thus, while the observed effects were more prominent in younger individuals, we cannot rule out that both age groups may have benefited equally from corrective information had our measures been more sensitive. Despite the age-related differences in scores and magnitude of our intervention effects, both age groups’ levels of belief in misinformation were relatively low in the current study. Intriguingly, our data show that even in cases of minimal misinformation belief, debunking strategies can be effective.

The last and possibly the most important finding from our study is the extent to which encountering misinformation after exposure to corrective misinformation diminishes the cognitive gains conferred by the latter. This finding is consistent with studies which discovered that strong misinformation messages “neutralised” the positive effects gained after exposure to communication about the consensus around climate change (Maertens et al., 2020). In the current social media context, these findings behoove public health agencies to consider how the already fleeting impact of light-touch interventions, such as mythbusters, might be further undercut by the very realistic prospect of subsequent exposure to misinformation. While it might be tempting to use these findings to call for corrective information to be delivered in a synchronized way between public health agencies and social media platforms, it is not clear how such strategies can be implemented on applications like WhatsApp where content is fully encrypted. These findings also call for more research examining the cognitive impact of such exposure to conflicting messages (i.e., corrective information followed by misinformation) on adherence to governmental directives (e.g., around preventive behaviors) among the public during infectious disease events. Thus far, we know that exposure to conflicting information around nutrition-related issues has been associated with nutrition confusion, backlash and decreased performance of healthy behaviors, such as fruit and vegetable consumption, and physical activity (Vijaykumar et al., 2021).

Further to the above discussions, it is worth highlighting the contribution of gender in the current study. We did not have a priori predictions surrounding gender or other demographic factors (e.g., employment) and, thus, did not control for gender distribution in our sampling. Still, inclusion of gender as a covariate revealed that males performed significantly poorer in our study, that is, they were more likely to deem misinformation statements to be more truthful and credible, and self-reported as being more likely to share the statements with others. Because of the unequal sampling of genders across age groups, we cannot draw heavily on these findings. Still, they do tentatively indicate that gender contributes to misinformation belief and behaviors, and that young males are at greater risk of believing in and sharing COVID-19 misinformation. Heightened susceptibility in young males resonates with existing work that has explicitly investigated the role of gender and other demographic and socioeconomic factors in COVID-19 misinformation belief (Pickles et al., 2021). Crucial to our findings, gender could not account for the discussed effects of age and our Intervention 2 manipulation. Building on these tentative findings to explore gender-specific misinformation effects would be a valuable avenue of future research.

What are the consequences of our findings? While not a natural experiment, our study design was premised on the fact that WhatsApp users can be exposed to the same misinformation once or multiple times from different sources in their small world network within a short period. In such a fast-moving informational environment, it would be inappropriate to classify corrective information as prebunking or debunking, given that it would be virtually impossible to determine who among millions of users have or have not already been exposed to misinformation. In this context, our findings show that the benefit of corrective information may be diminished if followed shortly by misinformation. This is especially pertinent given that misinformation research converges on the finding that “light-touch” interventions (such as single corrections, infographics, or “accuracy nudges”) are subject to rapid decay over time (Roozenbeek et al., 2021). Thus, our outcomes have major implications for implementing fact-check/accuracy nudges and other light-touch interventions in social media environments.

## Conclusion

Reinforcing exposure to corrective information could help maintain the gains from an initial dose of corrective information. While the exact number of repetitions required to maintain greater durability of corrective effects has yet to be understood, our findings suggest that a single dose of corrective information is insufficient. Existing work highlights the need for booster doses of corrective information. Still, our study is one of the few to demonstrate this need (a) in the context of the WHO’s official infographics and (b) among WhatsApp users. Moreover, our findings cut across younger and older adults, of whom the latter demonstrated a greater propensity to correctly identify misinformation and a lower tendency to share it. We suggest that public health agencies like the WHO leverage ongoing collaborations with the social media industry to ensure that users are repeatedly exposed to corrective information and gear these interventions among younger adults whose vulnerability to misinformation is becoming increasingly apparent.
